# Examining the feasibility of assisted index case testing for HIV case-finding: a qualitative analysis of barriers and facilitators to implementation in Malawi

**DOI:** 10.21203/rs.3.rs-3314925/v1

**Published:** 2023-09-08

**Authors:** Caroline J. Meek, Tiwonge E. Mbeya Munkhondya, Mtisunge Mphande, Tapiwa A. Tembo, Mike Chitani, Milenka Jean-Baptiste, Dhrutika Vansia, Caroline Kumbuyo, Katherine R. Simon, Sarah E. Rutstein, Clare Barrington, Maria H. Kim, Vivian F. Go, Nora E. Rosenberg

**Affiliations:** Gillings School of Global Public Health, University of North Carolina at Chapel Hill; Kamuzu University of Health Sciences; Baylor College of Medicine Children’s Foundation; Baylor College of Medicine Children’s Foundation; Baylor College of Medicine Children’s Foundation; Gillings School of Global Public Health, University of North Carolina at Chapel Hill; Baylor College of Medicine Children’s Foundation; Baylor College of Medicine Children’s Foundation; Baylor College of Medicine Children’s Foundation; Department of Medicine, Division of Infectious Diseases, University of North Carolina at Chapel Hill; Gillings School of Global Public Health, University of North Carolina at Chapel Hill; Baylor College of Medicine Children’s Foundation; Gillings School of Global Public Health, University of North Carolina at Chapel Hill; Gillings School of Global Public Health, University of North Carolina at Chapel Hill

**Keywords:** HIV testing and counselling, index case testing, assisted partner notification services, Malawi, implementation science, health care workers

## Abstract

**Background:**

Assisted index case testing, in which health care workers take an active role in referring at-risk contacts of people living with HIV for HIV testing services, has been widely recognized as an evidence-based intervention with high potential to increase PLHIV status awareness. Promising evidence for the approach has led to several attempts to scale assisted index case testing throughout eastern and southern Africa in recent years. However, despite effective implementation being at the heart of any assisted index case testing strategy, there is limited implementation science research from the perspective of the HCWs who are doing the “assisting”. This study examines the feasibility of assisted index case testing from the perspective of health care workers implementing the approach in Malawi.

**Methods:**

In-depth interviews were conducted with 26 lay health care workers delivering assisted index case testing in Malawian health facilities. Interviews explored health care workers’ experiences counselling index clients and tracing these clients’ contacts, aiming to inform development of a blended learning implementation package. Transcripts were inductively analyzed using Dedoose coding software to identify and describe key factors influencing feasibility of assisted index case testing. Analysis included multiple rounds of coding and iteration with the data collection team.

**Results:**

Participants reported a variety of barriers to feasibility of assisted index case testing implementation, including privacy concerns, limited time for assisted index case testing amid high workloads, poor quality contact information, logistical obstacles to tracing, and challenges of discussing sexual behavior with clients. Participants also reported several health care worker characteristics that facilitate feasibility: robust understanding of assisted index case testing’s rationale and knowledge of procedures, strong interpersonal skills, positive attitudes towards clients, and sense of purpose in their work.

**Conclusions:**

Findings demonstrate that maximizing assisted index case testing’s potential to increase HIV status awareness requires adequately equipping health care workers with appropriate knowledge, skills, and support to address and overcome the many feasibility challenges that they face in implementation.

**Trial Registration Number:**

NCT05343390

**Date of registration:** April 25, 2022

## Introduction

To streamline progress towards its goal of ending AIDS as a public health threat by 2030, the Joint United Nations Programme on HIV/AIDS (UNAIDS) launched a set of HIV testing and treatment targets [1]. Adopted by United Nations member states in June 2021, the targets call for 95% of all people living with HIV (PLHIV) to know their HIV status, 95% of all people with a diagnosed HIV infection to receive sustained antiretroviral therapy (ART) and 95% of all people receiving ART to achieve viral suppression by 2025 [2]. While many countries in eastern and southern Africa, including Malawi, have achieved the second and third targets related to treatment and viral suppression, as of 2022 no country in the region had met the first target related to awareness of HIV status in PLHIV [3]. As 2025 approaches, strategies are needed to address the first 95% target.

Index case testing (ICT), which facilitates targeted provision of HIV testing services (HTS) for sexual partners, biological children, and other contacts of known PLHIV (“index clients”), is a promising strategy to accelerate progress towards the PLHIV status awareness target [4, 5]. Traditional approaches to ICT rely on passive referral, in which index clients invite their contacts for testing [6]. However, the World Health Organization (WHO) and the President’s Emergency Plan for HIV/AIDS Relief (PEPFAR) have both recommended assisted approaches to ICT [7, 8, 9], in which health care workers (HCWs) take an active role in referral of at-risk contacts for testing, due to improved effectiveness compared to passive approaches [10, 11, 12, 13, 14, 15, 16, 17]. As a result, there have been attempts to scale assisted ICT throughout southern and eastern Africa over the past several years. Implementation evidence [18] from the region suggests that assisted ICT can be an acceptable [6, 17, 19, 20, 21, 22, 23], cost-effective [24], and low-risk [25] strategy to promote PLHIV status awareness [26, 27, 28, 29]. However, despite effective implementation being at the heart of any assisted ICT strategy, there is limited implementation science research from the perspectives of the HCWs who are doing the “assisting”. Particularly, further study is needed of the feasibility, or the extent to which an intervention can be successfully carried out within a given setting [18], of assisted ICT from the perspective of HCWs.

This qualitative analysis addresses this gap by exploring factors influencing the feasibility of assisted ICT from the HCW perspective. The aim of this analysis was to provide formative insights into how to best support assisted ICT delivery in the context of an implementation science trial in Malawi.

## Methods

### Setting

This study was conducted in the Machinga and Balaka districts of Malawi. Malawi is a country in southeastern Africa in which 8.9% of adults are living with HIV and an estimated 88.3% of these adults are aware of their HIV-positive status [30]. Malawi has a long-established passive ICT program, and in 2019 the country also adopted an assisted component, known as voluntary assisted partner notification, as part of its national HIV testing policy [31]. While Malawi has one of the lowest rates of qualified HCWs globally [32], the country has a strong track record of shifting HTS tasks to lay HCWs with less formal training in order to mitigate this limited medical workforce capacity [33]. In particular, Malawi’s Tingathe Program has harnessed lay HCW capacity to rapidly and efficiently scale up HTS [34, 35, 36], offering a variety of assisted ICT approaches including 1) contract referral, in which HCWs follow up with contact clients who do not visit the facility for HTS by an agreed-upon date; 2) provider referral, in which HCWs locate and notify contact clients while maintaining the anonymity of index clients; and 3) dual referral, in which HCWs partner with index clients to facilitate couples HIV testing and status disclosure [6].

### Study design

Data for this analysis were collected as part of formative research for a two-arm cluster randomized control trial examining a blended learning implementation package as a strategy for building HCW capacity in assisted ICT. Earlier work [31] established the theoretical basis for testing the blended learning implementation package, which combines individual asynchronous modules with synchronous small-group interactive sessions to enhance training and foster continuous quality improvement. The formative research presented in this paper aimed to further explore factors influencing feasibility of the assisted ICT from the perspective of HCWs in order to identify areas on which to focus training content and problem-solving discussions.

Prior to the start of the trial (October-December 2021), the research team conducted 26 in-depth interviews (IDIs) with lay HCWs at 14 facilities. These 14 facilities were selected from the 34 facilities included in the trial and we purposively selected facilities in both districts at different types of facilities (hospitals, health centers, and dispensaries). To be eligible for participation in the parent study, HCWs had to be at least 18 years old, work full-time at one of the health facilities included in the study, and be involved in counselling index clients and/or tracing their contacts.

### Data collection

IDIs were conducted by three trained Malawian interviewers in a private setting using a semi-structured guide. IDIs were conducted over the phone when possible (n = 18) or in-person at sites with limited phone service (n = 8). The semi-structured guide was developed through a series of rigorous, iterative discussions among the research team. The questions used for this analysis were a subset of a larger interview. The interview guide questions for this analysis explored HCWs’ experiences with assisted ICT, including barriers and facilitators to implementation. Probing separately about the processes of counselling index clients and tracing their contacts, interviewers asked questions such as “What is the first thing that comes to mind when you think of counselling index clients/tracing contacts?”, “What aspects do you [like/not like] about…?” and “What do your colleagues say about…?”. When appropriate, interviewers probed further about how specific factors mentioned by the participant facilitate or impede the ICT implementation experience.

The IDIs lasted from 60–90 minutes and were conducted in Chichewa, a local language in Malawi. Eleven audio recordings transcribed verbatim in Chichewa before being translated into English and 15 recordings directly translated and transcribed into English. Interviewers summarized each IDI after it was completed, and these summaries were discussed with the research team routinely.

### Data analysis

The research team first reviewed all of the interview summaries individually and then met multiple times to discuss initial observations, refining the research question and scope of analysis. A US-based analyst (CJM) with training in qualitative analysis used an inductive approach to develop a codebook, deriving broad codes from the implementation factors mentioned by participants throughout their interviews. Along with focused examination of the transcripts, she consulted team members who had conducted the IDIs with questions or clarifications. CJM regularly met with Malawian team members (TEMM, MM, TAT) who possess the contextual expertise necessary to verify and enhance meaning. She used the Dedoose (2019) web application to engage in multiple rounds of coding, starting with codes representing broad implementation factors and then further refining the codebook as needed to capture the nuanced manifestations of these barriers and facilitators. Throughout codebook development and refinement, the analyst engaged in memoing to track first impressions, thought processes, and coding decisions. The analyst presented the codebook and multiple rounds of draft results to the research team. All transcripts and applied codes were also reviewed in detail by additional team members (MJB, DV). Additional refinements to the codebook and results interpretations were iteratively made based on team feedback.

### Ethical clearance

Ethical clearance was provided by UNC’s IRB, Malawi’s National Health Sciences Research Committee, and the Baylor College of Medicine IRB. Written informed consent was obtained from all participants in the main study and interviewers confirmed verbal consent before starting the IDIs.

## Results

Participant roles included community HCWs and counsellors. Eight were females and eighteen were males. Sixteen participants were from Machinga and ten were from Balaka. Two of the facilities were district hospitals, eight were health centers, and three were dispensaries.

### Factors influencing feasibility of assisted ICT: barriers and facilitators

Participants described many barriers to assisted ICT, including privacy concerns, limited time for ICT amid high workloads, poor quality contact information, logistical obstacles to tracing, and challenges of discussing sexual behavior with clients. In addition to these barriers, participants also described several HCW characteristics that facilitated feasibility: ICT knowledge, interpersonal skills, positive attitudes towards clients, and sense of purpose. Each of these is described in greater detail:

### Feasibility Barriers

#### Privacy concerns

Concerns about privacy in the clinic setting were identified as a determinant of index clients’ openness and honesty with HCWs about their contacts. They expressed that index and contact clients, largely driven by fear of unwanted disclosure due to the intense stigma surrounding HIV, highly value privacy. Participants frequently discussed clinics’ physical layout, indicating that counselling index clients is more successful when conducted in private settings where others cannot overhear personal information. Not all clinics have individual rooms available for private conversation; when available, these rooms were often at high risk of interruption by other staff due to high demand. One participant described this challenge: “*if I’m counseling an index client and people keep coming into the room…this compromises the whole thing because the client becomes uncomfortable in the end. When a client is trying to respond to a question and someone comes in, the client is disturbed and might forget what [they] wanted to say, might even feel shy.*” Some HCWs mentioned working around this issue through use of screens, “do-not-disturb” signs, outdoor spots, and tents.

Privacy in the community setting was also highlighted as an important factor in contact clients’ engagement with HCWs and willingness to be tested for HIV. Participants noted that it can be particularly challenging to maintain privacy when tracing contact clients in the field, as they sometimes find clients in a situation that is not conducive to sensitive conversations. One participant described: “*we get to the house and find that there are 4, 5 people with our [contact client]…it doesn’t go well…That is a mission gone wrong*.” Participants also noted that HCWs are also often easily recognizable in the community due to their Buffalo-brand bikes, uniforms, and cars, which exacerbates the risk of compromising privacy. To address privacy challenges in the community, participants reported strategies to increase discretion, including dressing to blend in with the community, preparing an alternate reason to be looking for the client, and testing multiple people or households to avoid singling out one person.

#### Limited time for ICT amid high workloads

Some participants indicated that strained staffing capacity leads HCWs to have to perform multiple roles, expressing challenges in balancing their ICT work with their other tasks. As one participant described, *“We are as 3, since we are both the community health workers and counselors [and] we are supposed to be tracing.”* Some also described being the only, or one of few staff responsible for ICT: *“You’re doing this work alone, so you can see that it is a big task to do it single-handedly.”* The need to counsel each index client individually, as a result of confidentiality concerns, further increases workload for the limited staff assigned to this work. Multiple HCWs noted the need to hire more staff dedicated to ICT work.

High workloads also resulted in shorter appointments and less time to counsel index clients, which participants reported limits the opportunity for rapport that facilitates openness or probes for detailed information about sexual partners. Participants emphasized the importance of having enough time to meaningfully engage with index clients: *“For counselling you cannot have a limit to say, ‘I will talk to him for 5 minutes only.’ …That is not counselling then. You are supposed to stay up until…you feel that this [person] is fulfilled.”*. In addition, high workload can reduce the capacity of HCWs to deliver quality counseling: *“So you find that as you go along with the counseling, you can do better with the first three clients but the rest, you are tired and you do short cuts.”*

High workloads also lead to longer queues, which may deter clients from coming into the clinic or cause them to leave before receiving counselling: *“Sometimes because of shortage of staff, it happens that you have been assigned a certain task that you were supposed to do but at the same time there are clients who were supposed to be counseled. As a result, because you spent more time on the other task as a result you lose out some of the clients because you find that they have gone.”* Some participants emphasized the need to make HTS and ICT as easy and convenient as possible.

#### Poor quality contact information

Participants repeatedly discussed the importance of eliciting accurate information about a person’s sexual partners, including where, when, and how to best contact them. As one participant said, “*Once the index has given us the wrong information then everything cannot work, it becomes wrong…if he gives us full information [with] the right details then everything becomes successful and happens without a problem*.” Adequate information is a critical component of the ICT process, as incorrect or incomplete information delays or prevents communication with contact clients.

Inadequate information, which can include incorrect or incomplete names, phone numbers, physical addresses, and contextual details, can arise from a variety of scenarios. Most participants mentioned index clients providing incorrect information as a concern. This occurred either intentionally to avoid disclosure or unintentionally if information was not known. Intentionally false information was portrayed as a particular risk with newly diagnosed index clients who are still grappling with the news of their diagnosis. Poor quality contact information also results from insufficient probing and poor documentation, which is often exacerbated by aforementioned HCW time and energy constraints. In one participant’s words, *“The person who has enlisted the contact…is the key person who can make sure that our tracing is made easy.”* Participants noted the pivotal role of the original HCW who first interacts with the index client in not only eliciting correct locator information but also eliciting detailed contextual information. For example, details about a contact client’s profession are helpful to trace the client at a time when they will likely be at home. Other helpful information included nicknames, HIV testing history, and notes about confidentiality concerns.

#### Logistical obstacles to tracing

Some contact clients are reached by phone whereas others must be physically traced in the community. Some participants reported difficulty with tracing via phone, frequently citing network problems and lack of sufficient airtime allocated by the facility. Participants also reported that some clients were unreachable by phone, necessitating physical tracing. Physically tracing a contact client requires a larger investment of resources than phone tracing, especially when the client lives at a far distance from the clinic. Participants frequently discussed having to travel far distances to reach contact clients, an issue some saw as exacerbated by people who travel to clinics at far distances due to privacy concerns.

While most participants reported walking or biking to reach contact clients in the community, some mentioned using a motorcycle or Tingathe vehicle. However, access to vehicles is often limited and these transportation methods require additional expenses for fuel. Walking or biking was also reported to expose HCWs to inclement weather, including hot or rainy seasons, and potential safety risks such as violence.

Participants reported that traveling far distances can be physically taxing and time-consuming, sometimes rendering them too tired or busy to attend to other tasks. Frequent travel influenced HCW morale, particularly when a tracing effort did not result in successfully recruiting a contact client. Participants frequently described this perception of wasted time and energy as “*painful*”, with the level of distress often portrayed as increasing with the distance travelled. As one HCW said, *“You [can] find out that he gave a false address. That is painful because it means you have done nothing for the person, you travelled for nothing.”*

HCWs described multiple approaches used to strategically allocate limited resources for long distances. These approaches included waiting to physically trace until there are multiple clients in a particular area, reserving vehicle use for longer trips, and coordinating across HCWs to map out contact client locations. HCWs also mentioned provision of rain gear and sun protection to mitigate uncomfortable travel. Another approach involved allocating contact tracing to HCWs based in the same communities as the contact clients.

#### Challenges of discussing sexual behavior with clients

Participants described difficulties in approaching index clients about their sexual history, particularly youth and newly diagnosed index clients. Youth were reported to be *“in a hurry”, “shy”*, *“[thinking] they know a lot”,* and *“difficult to reveal their contacts.”* Participants described newly diagnosed index clients as often having trouble accepting and processing an HIV diagnosis, and as a result may be unable to engage in counselling.

When asked about contact clients that are difficult to trace, participants often mentioned male sexual partners of index clients. Men sometimes excessively question HCWs about who referred them for HTS, delay or refuse HTS. In addition, angry or threatening responses were more frequently reported in men, who often associated clinic staff with HIV and perceived questions about sexual behavior as invasive or accusatory. A few participants recounted stories of being physically threatened by angry contact clients. Participants also reported that HIV-discordance can create acute conflict in couples, creating uncertainty for HCWs. Some participants verbalized concern about index client risk of intimate partner violence (IPV) upon partner disclosure, particularly for women.

### Feasibility Facilitators

#### HCW knowledge about ICT

Participants reported that HCWs with a thorough understanding of ICT’s rationale and purpose can facilitate client openness. Clients were more likely to engage with HCWs about assisted ICT if they understood the benefits to themselves and their loved ones. One HCW stated, *“If the person understands why we need the information, they will give us accurate information.”*

Participants also discussed the value of deep HCW familiarity with ICT procedures and processes, particularly regarding screening clients for IPV and choosing referral method. One participant described the importance of clearly explaining various referral methods to clients: *“So…people come and choose the method they like…when you explain things clearly it is like the index client is free to choose a method which the contact can use for testing”.* Thorough knowledge of available referral methods allows HCWs to actively engage with index clients to discuss strategies to refer contacts in a way that fits their unique confidentiality needs, which was framed as particularly important when IPV is identified as a concern. Multiple participants suggested the use of flipcharts or pamphlets, as well as regular opportunities for training, to continuously “refresh” their ICT knowledge in order to facilitate implementation.

#### HCW interpersonal skills

In addition, HCWs’ ability to navigate sensitive conversations about sexual health was noted as a key facilitator of successful implementation. Interpersonal skills were mentioned as mitigating the role’s day-to-day uncertainty by preparing HCWs to engage with clients, especially newly diagnosed clients: “*I need to counsel them skillfully so that they understand what I mean regardless that they have just tested positive for HIV.*”

When discussing strategies to build HCW skills in counselling index clients and tracing contact clients, participants suggested establishing regular opportunities to discuss challenges and share approaches to address these challenges: “*I think that there should be much effort on the [HCWs] doing [ICT]. For example, what do I mean, they should be having a meeting with the facility people to ask what challenges are you facing and how can we end them?”*. Another participant further elaborated, saying *“We should be able to share experiences with our [colleagues] so that we can all learn from one another. And also, there are other people who are really brilliant at their job. Those people ought to come visit us and see how we are doing. That is very motivating.”*

#### HCW positive attitudes towards clients

Participants also highlighted the role of empathy and non-judgement in building trust with clients: “*Put yourself in that other person’s shoes. In so doing, the counseling session goes well. Understanding that person, that what is happening to them can also happen to you*.”. Participants viewed trust-building as critical to facilitating client comfort and openness: “*if they trust you enough, they will give you the right information.”* Further, participants associated HCW assurance of confidentiality with promoting trust and greater information sharing: “*Also assuring them on the issue of confidentiality because confidentiality is a paramount. If there will not be confidentiality then the clients will not reveal.”*

#### HCW sense of purpose

Lastly, several participants reported that a sense of purpose and desire to help people motivated them to overcome the challenges of delivering assisted ICT. One participant said, “*Some of these jobs are a ministry. Counseling is not easy. You just need to tell yourself that you are there to help that person*.” Many seemed to take comfort in the knowledge that their labors, however taxing, would ultimately allow people to know their status, take control of their health, and prevent the spread of HIV. Participants framed the sense of fulfillment from successful ICT implementation as a mitigating factor amidst challenges: “*If [the contact client] has accepted it then I feel that mostly I have achieved the aim of being in the health field…that is why it is appealing to me*”.

## Discussion

Participants identified several important barriers to ICT, including privacy concerns, limited time for ICT amid high workloads, poor quality contact information, challenges of physical tracing, and complexities of discussing sexual behavior with clients. However, participants also described several key HCW characteristics that can help mitigate these barriers to promote feasibility, such as knowledge, skills, and attitudes.

Several of the barriers and facilitators we identified were intimately interconnected. Perceptions of privacy, time constraints, and HCW characteristics (knowledge, skills, and attitudes) all contribute to the extent to which counselling index clients elicits adequate information to facilitate contact tracing. Information quality has implications for HCW capacity, as inadequate information can lead to wasted resources, including HCW time and energy, on contact tracing. The opportunity cost of wasted efforts, which increases as the distance from which the contact client lives from the clinic increases, depletes HCW morale. The resulting acceleration of burnout, which is already fueled by busy workloads and the inherent uncertainty of day-to-day ICT work, further impairs HCW capacity to effectively engage in quality counseling that elicits adequate information from index clients.

Our study is among the first to provide HCW perceptions of assisted ICT. The studies that do exist in the region similarly characterize the HCW experience of delivering assisted ICT as challenging due to inadequate client information sharing arising from confidentiality concerns and anticipated stigma, time constraints, large resource investments required for tracing work, challenges in engaging with clients about sexual behavior [20, 21, 22, 37]. These studies also emphasize effective HCW training in ICT processes, confidentiality assurance, and non-judgmental rapport-building; as well as sufficient infrastructure, transportation, and communication support; as critical components of successful assisted ICT implementation. Cumulative evidence of barriers across different settings suggests that assisted ICT implementation may pose greater burden on HCWs than previously thought [6], but also suggests that strategic investment in HCW training and support has the potential to help overcome these barriers.

Our findings further inform the types of implementation strategies that may enhance assisted ICT, an important need. In our own work, these findings affirmed the rationale for the blended learning implementation package tested in our trial. Participants expressed that promoting HCW knowledge and skills can mitigate the many barriers to feasibility of assisted ICT that they face, with some highlighting the value of learning from others’ experiences and some suggesting the need for opportunities to refresh their knowledge and skills over time. The blended learning implementation package addresses this balance by providing time for HCWs to master ICT knowledge with a combination of in-person sessions (which allow for practicing and feedback) and asynchronous, digitally delivered content (which allows for continuous review). In addition, participants frequently mentioned informal workarounds currently in use to mitigate barriers or offered up ideas for potential solutions to try. Our blended learning implementation package streamlines these problem-solving processes by offering monthly continuous quality improvement sessions at each facility in our enhanced arm. These sessions allow for structured time to discuss identified barriers as well as how to efficiently allocate limited resources in their specific setting.

In addition, findings informed training content and priority areas for quality improvement. For example, our training package includes a detailed description of each referral method and emphasizes client-centered, non-judgmental counseling as a way to mitigate stigma and improve outcomes. Focus areas for continuous quality improvement discussions include use of space, staffing, allocation of airtime and vehicles, and documentation.

## Conclusions

Assisted ICT has been widely recognized as an intervention with high potential to increase PLHIV status awareness [6, 13, 14, 17, 19, 20, 21, 22, 23, 24, 25, 26, 28, 29], which is important as countries in eastern and southern Africa strive to reach global UNAIDS targets. This study examines the implementation of assisted ICT from the perspective of very people doing the “assisting.” While HCWs view assisted ICT as a valuable strategy to increase HIV status awareness, they face a range of barriers to feasibility that hinder successful implementation. Findings demonstrate that maximizing assisted ICT’s potential requires adequately equipping HCWs with appropriate knowledge, skills, and support to address and overcome the many challenges that they face in implementation.

## Data Availability

Qualitative data on which this analysis is based, as well as data collection materials and codebooks, are available from the last author upon reasonable request.

## Abbreviations

AIDS: Acquired Immunodeficiency Syndrome
ART: Antiretroviral Therapy
HCW: Health Care Worker
HIV: Human Immunodeficiency Virus
HTS: HIV Testing Services
ICT: Index Case Testing
IDI: In-Depth Interview
IPV: Intimate Partner Violence
IRB: Institutional Review Board
PEPFAR: President’s Emergency Plan for HIV/AIDS Relief
PLHIV: People Living With HIV
UNAIDS: Joint United Nations Programme on HIV/AIDS
WHO: World Health Organization
